# Effect of Different Anthocyanidin Glucosides on Lutein Uptake by Caco-2 Cells, and Their Combined Activities on Anti-Oxidation and Anti-Inflammation In Vitro and Ex Vivo

**DOI:** 10.3390/molecules23082035

**Published:** 2018-08-14

**Authors:** Minh Anh Thu Phan, Martin Bucknall, Jayashree Arcot

**Affiliations:** 1 Food and Health Cluster, School of Chemical Engineering, UNSW, Sydney, NSW 2052, Australia; 2 Mark Wainwright Analytical Centre, UNSW, Sydney, NSW 2052, Australia; m.bucknall@unsw.edu.au

**Keywords:** anthocyanins, lutein, interaction, cellular antioxidant activity, anti-inflammatory activity, cellular uptake interference

## Abstract

The interactive effects on anti-oxidation and anti-inflammation of lutein combined with each of the six common anthocyanidin glucosides were studied in both chemical and cellular systems. The combined phytochemicals showed an antagonism in the inhibition of lipid oxidation in a liposomal membrane, but showed an additive effect on cellular antioxidant activity in Caco-2 cells. Lutein was an active lipoxygenase inhibitor at 2–12 μM while anthocyanins were inactive. The concentration of lutein when it was used in combination with anthocyanins was 25–54% higher than when lutein was used alone (i.e., IC_50_ = 1.2 μM) to induce 50% of lipoxygenase inhibition. Only the combination of lutein with malvidin-3-glucoside showed anti-inflammatory synergy in the suppression of interleukin-8, and the synergy was seen at all three ratios tested. Some mixtures, however, showed anti-inflammatory antagonism. The presence of anthocyanins (5–7.5 μM) did not affect lutein uptake (2.5–5 μM) by Caco-2 cells.

## 1. Introduction

Lutein is a xanthophyll carotenoid mainly present in dark green leafy vegetables [1]. Lutein shows antioxidant and anti-inflammatory activities by targeting reactive oxygen species, and downregulating inflammatory proteins and pro-inflammatory cytokines [2]. Lutein is one of the three xanthophyll carotenoids that can cross the blood-brain barrier and selectively accumulate in the retina and brain tissues [1,3,4]. The xanthophyll lutein is often co-ingested with other plant phytochemicals such as carotenoids and/or flavonoids in a normal human diet containing plant-based foods. Anthocyanins are one of the largest classes of flavonoids and are present abundantly in many fruits and vegetables [5]. Thus, there are chances for lutein and anthocyanins to be concurrently consumed in a meal, after which they can interact with each other during digestion and absorption to effect biological activities. Water-soluble phytochemicals may interfere with the uptake of lipid-soluble bioactive compounds [6]. For instance, lutein uptake by Caco-2 cells is impaired by the flavonoid naringenin, but is not affected by (+)-catechin, a phenolic acid, or vitamin C [1]. Absorption interference between phytochemicals may result in changes on combined biological effects of the compounds [6]. We have published a paper reporting that anthocyanins increase β-carotene uptake by Caco-2 cells to levels that trigger β-carotene’s pro-oxidant activity, which results in an antagonistic cellular antioxidant effect seen in some combinations [7]. Phytochemical interactions on cellular uptake and biological activities are often studied separately, so the mutual influences between these aspects are not well addressed. This study aimed to investigate the effect of different common anthocyanidin glucosides on lutein uptake by Caco-2 cells, and the combined effects of anthocyanins and lutein on oxidative inhibition and anti-inflammation in both chemical and cellular models.

## 2. Results and Discussion

### 2.1. Effects of Lutein-Anthocyanin Combinations on Oxidative Inhibition in Chemical and Cellular Models

#### 2.1.1. Inhibitory Effect on Liposome Peroxidation

**Figure 1:**
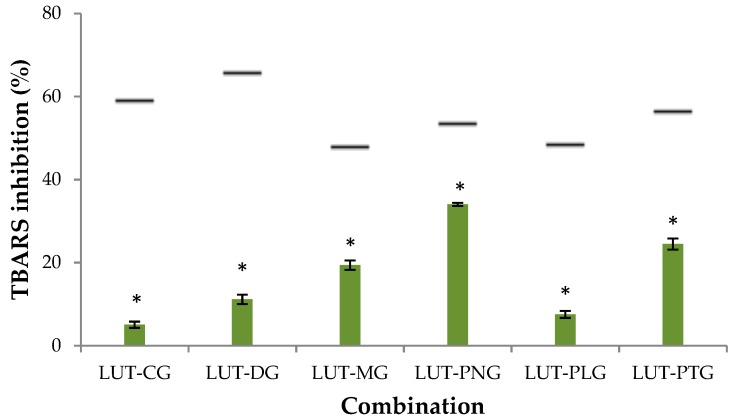
Liposome peroxidation inhibitory activity of different lutein-anthocyanin combinations (1:1 ratio). The horizontal lines illustrate the expected additive effect of lutein-anthocyanin combinations. Asterisk-marked columns indicate a significant difference (*p* < 0.05) between the observed effect of the mixture with its calculated additive effect. Calculation of the expected additive effect was based on an equation of Fuhrman et al. [12]: TBARS_A_ + TBARS_L_ − TBARS_A_ × TBARS_L_/100 (TBARS_A_ and TBARS_L_ are %TBARS inhibition of anthocyanin alone and lutein alone respectively), which was calculated following an equation given in [7]. TBARS: thiobarbituric acid reagent species, LUT: lutein, CG: kuromanin chloride, DG: myrtillin chloride, MG: oenin chloride, PNG: peonidin-3-glucoside chloride, PLG: callistephin chloride, PTG: petunidin-3-glucoside chloride.

The percentage of thiobarbituric acid reagent species (%TBARS) inhibition when lutein was present alone was 39%, and when anthocyanins were present alone, %TBARS inhibition was 14–43%. Lutein combined with each of the tested anthocyanins did not enhance the inhibitory effect on lipid peroxidation in the liposomal membrane. The expected additive effects of TBARS inhibition of lutein-anthocyanin mixtures were 48–66%, but the actual effects of the mixtures were less than 35% (Figure 1). This indicates that lutein and anthocyanins showed an antagonistic interaction at the interface of the liposomal membrane. Lutein is a xanthophyll carotenoid characterized with polar groups at the two ends of its molecule. Lutein can position itself in parallel closely to the polar heads of the membrane, or it can span the molecule across the membrane with the polar ends anchoring to the polar lipid heads [8]. Anthocyanin compounds are normally positioned in the aqueous region of the membrane outer monolayer [9]. Such orientations of the compounds in the lipid bilayer membrane may enable them to interact and form lutein-anthocyanin adducts, which result in the reduced capability of lipid peroxidation inhibition. The formation of adducts between other carotenoids and flavonoids, for example: β-carotene and green tea polyphenolic compounds [10] or β-carotene and daidzein [11], has been previously reported to impart antioxidant antagonism in liposomes.

#### 2.1.2. Cellular Antioxidant Activity (CAA)

The interactive effects on anti-oxidation of lutein-anthocyanin combinations at 1:1, 1:3 and 3:1 ratios were assessed in a Caco-2 cell model. There was no synergistic or antagonistic effect seen in any of the mixtures at the tested ratios. All combinations showed additive CAA in Caco-2 cells (Table 1). Lutein and the anthocyanidin glucosides showed antagonistic interaction in the phosphatidylcholine (PC) liposome membrane, but did not show the same interaction in the cell membrane. The different interactions between phytochemicals can be seen in different assay models [6,13]. A combination of phytochemicals may show synergy/antagonism in chemical models, but may not show the same in cellular models, and vice versa. For example, the combination of raspberry and adzuki bean extracts showed antioxidant synergy in chemical assays but did not show the same effect in MCF-7 cancerous cells [14]. On the other hand, membrane lipid composition has a pronounced effect on the localization of phytochemicals and the interaction of the phytochemicals with the membrane, which may lead to changes in biological activities [15]. The interactive effect of lutein and anthocyanins in the PC liposome membrane being different from that in the Caco-2 cell membrane might be partly due to the differences in the composition of the two membrane models.

### 2.2. Effects of Lutein-Anthocyanin Combinations on Anti-Inflammation in Chemical and Cellular Models

#### 2.2.1. Lipoxygenase Inhibitory Activity

**Figure 2:**
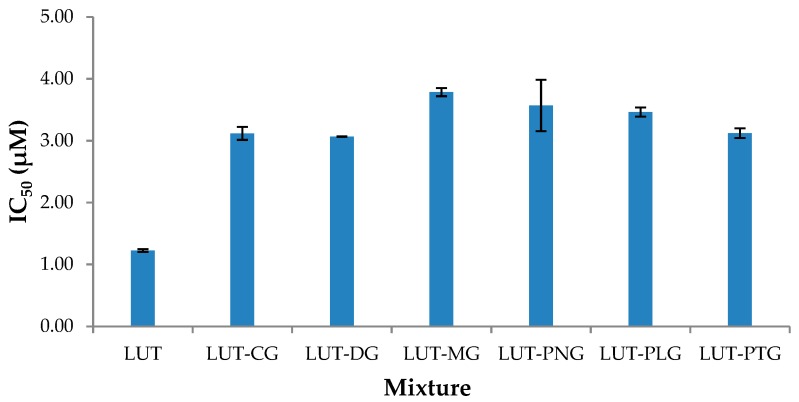
Lipoxygenase IC_50_ of lutein alone and different lutein-anthocyanin combinations. IC_50_: inhibitory concentration that exerts 50% enzyme inhibition. LUT: lutein, CG: kuromanin chloride, DG: myrtillin chloride, MG: oenin chloride, PNG: peonidin-3-glucoside chloride, PLG: callistephin chloride, PTG: petunidin-3-glucoside chloride.

Lutein showed strong inhibition of LOX-1 (IC_50_ = 1.2 μM). None of the anthocyanins showed potent LOX-1 inhibitory activity at 2–12 μM (% LOX-1 inhibition of 0.5–12.3%). They have been reported to have high LOX-1 IC_50_, for example: peonidin-3-glucoside (PNG): 38 mM, or kuromanin chloride (CG): 0.5 mM [16]. The mode of interactive effect upon LOX-1 inhibition between lutein and anthocyanins could not be determined because lutein was an active LOX-1 inhibitor at low concentrations while anthocyanins were not. The lipoxygenase inhibitory effects of all lutein-anthocyanin mixtures were still measured to evaluate whether the presence of anthocyanins affected the LOX-1 inhibitory activity of lutein. IC_50_ values of lutein-anthocyanin mixtures ranged from 3.1–3.8 μM, which were higher than that of the single lutein (IC_50_ = 1.2 μM) (Figure 2). This indicates that lutein combined with anthocyanins inhibited LOX-1 less effectively than lutein alone. The concentrations of lutein required for the mixtures to exhibit 50% of LOX-1 inhibition were increased by 25–54% of the IC_50_ of lutein when it was applied alone. These results show that the presence of anthocyanins affected the LOX-1 inhibitory activity of lutein. Anthocyanins and carotenoids inhibit LOX-1 non-competitively [16,17] by binding to the lipoxygenase-substrate complex. The reduced LOX-1 inhibitory effect of lutein when it was present with anthocyanins might be due to the interference of the anthocyanins with the binding of lutein to the lipoxygenase-substrate complex.

#### 2.2.2. Secretion of Interleukin-8 (IL-8)

**Figure 3:**
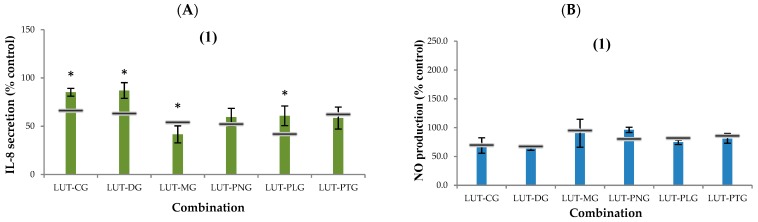

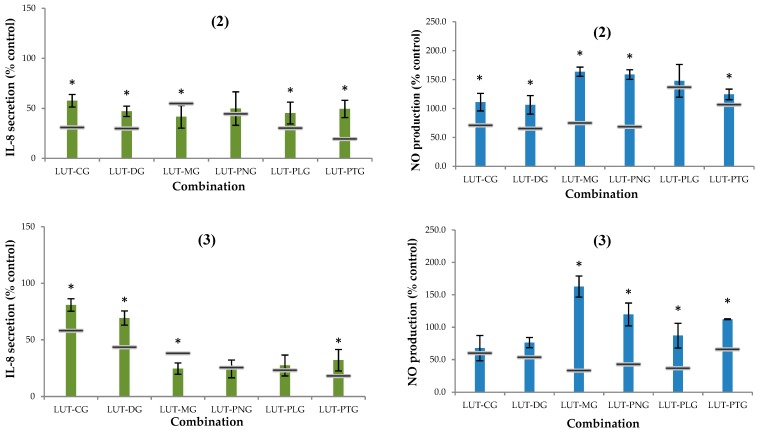
Secretion of (**A**) IL-8 (% control) and (**B**) nitric oxide (NO) (% control) by Caco-2 cells after being treated with different lutein-anthocyanin mixtures followed by TNF-α-induced inflammation (100 ng/mL). Total concentration of the bioactive compounds in cell culture was 10 μM and the ratio of lutein to anthocyanin was varied at (1) 1:3, (2) 1:1, and (3) 3:1. Controls are samples collected from the cells that underwent TNF-α-induced inflammation without pre-treatment with phytochemicals. The horizontal lines illustrate the expected additive effect, which was calculated as 100 − (A + L − A × L/100) (A and L are % reduction of IL-8 or NO secreted by Caco-2 cells compared to the control when treating the cells with anthocyanin alone and lutein alone respectively). Asterisk-marked columns indicate a significant difference (*p* < 0.05) between the observed effect of the combination with its calculated additive effect. Experimental values show as mean ± SD of three independent replicates. LUT: lutein, CG: kuromanin chloride, DG: myrtillin chloride, MG: oenin chloride, PNG: peonidin-3-glucoside chloride, PLG: callistephin chloride, PTG: petunidin-3-glucoside chloride.

The % interleukin-8 secretion compared to the control when lutein (2.5, 5, 7.5 μM) was applied alone was 78%, 70% and 57%, respectively. Most of the lutein-anthocyanin mixtures effectively reduced the amount of IL-8 secreted by Caco-2 cells after TNF-α-induced inflammation. The effectiveness of suppressing IL-8 secretion when lutein was combined with CG or myrtillin chloride (DG) was lower than when it was combined with the other anthocyanins. The mixtures of lutein with oenin chloride (MG), PNG, callistephin chloride (PLG) or petunidin-3-glucoside (PTG) increasingly reduced IL-8 secretion when the ratio of lutein to anthocyanins was increased (Figure 3). The lutein-oenin chloride combination (LUT-MG) was the only combination that showed a synergistic effect on interleukin-8 suppression, and the synergy was seen at all three ratios tested. The LUT-PNG mixture showed an additive effect at all three tested ratios, and some mixtures showed an antagonistic effect, including: LUT-CG and LUT-DG at all three ratios tested; LUT-PLG at the lutein:anthocyanin ratios of 1:3 and 1:1; and LUT-PTG at the 1:1 and 3:1 ratios.

#### 2.2.3. Nitric Oxide (NO) Production

**Figure 4:**
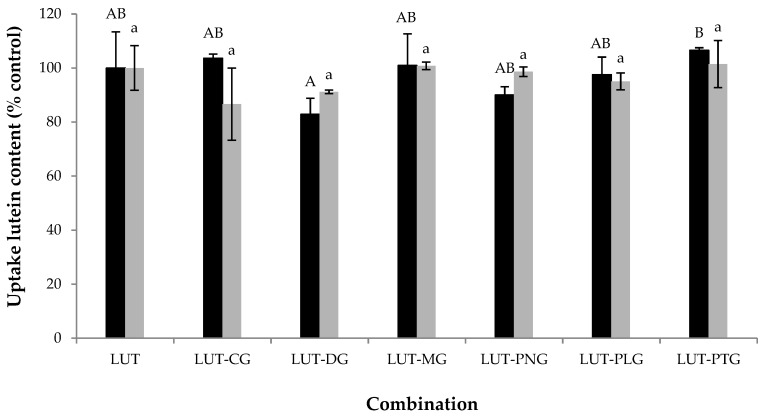
Lutein uptake (% control) by Caco-2 cells in the absence (control) and presence of different anthocyanins at lutein:anthocyanin ratios of 1:3 (2.5:7.5 μM, represented in black bars) and 1:1 (5:5 μM, represented in grey bars). Columns of the same colour marked with different letters indicate a significant difference from each other (*p* < 0.05). Values are mean ± SD of three independent replicates. LUT: lutein, CG: kuromanin chloride, DG: myrtillin chloride, MG: oenin chloride, PNG: peonidin-3-glucoside chloride, PLG: callistephin chloride, PTG: petunidin-3-glucoside chloride.

The % NO production compared to the control when lutein (2.5, 5, 7.5 μM) was applied alone was 95%, 81% and 76%, respectively. Most of the combinations of lutein with anthocyanins did not effectively inhibit the production of nitric oxide (Figure 4). Synergy was not seen in any of the mixtures. An antagonistic effect was observed in most of the combinations at the 1:1 and 3:1 ratios of lutein to anthocyanins. All mixtures showed an additive effect at the lutein:anthocyanin ratio of 1:3.

### 2.3. Interferences of Anthocyanins on Lutein Uptake by Caco-2 Cells

The cellular uptake of lutein (5 μM) in the presence of each of the tested anthocyanins (5 μM) was not significantly different (*p* > 0.05) from the lutein uptake when it was present alone (Figure 3). The same trend was observed when the ratio of anthocyanin to lutein was increased to 7.5 μM:2.5 μM (Figure 3). These results indicate that anthocyanins did not affect the uptake of lutein by Caco-2 cells. The effects of some polyphenols on lutein uptake by Caco-2 cells have been previously reported. (+)-catechin, gallic acid and caffeic acid do not affect the cellular absorption of lutein, whereas naringenin causes an impairment of lutein uptake [1]. The latter has been suggested to be the consequence of the interaction of naringenin with the membrane lipids, which influences the invagination of the lipid raft domains containing lutein receptors [1]. Anthocyanins can incorporate into the polar interface of the membrane outer monolayer [9] leading to an increase in the polarization area, which may result in a mismatch between the area of the polar heads and the area of the hydrophobic tails [18]. Consequently, the interspace between the two lipid layers can be increased, giving additional freedom to the hydrocarbon chains. This effect is called membrane fluidization, which may influence the appearance and development of lipid rafts (the so-called raft-breaking effect [18]), leading to a reduced diffusion of some lipid molecules. Membrane fluidization, on the other hand, decreases lipid-melting temperatures which possibly results in an increase in lipid diffusion [18]. These contradictory effects of polar flavonoids upon the diffusion of lipophilic molecules were seen in anthocyanins affecting the uptake of carotenoids. We previously reported that some anthocyanins (7.5 μM) increase β-carotene uptake (2.5 μM) [7]. These anthocyanin compounds, however, decreased lycopene absorption (data not shown) and did not influence lutein uptake. It seems that the interaction of anthocyanins with the cellular lipid membrane did not affect the lipid raft domains that contain lutein receptors.

The combinations of lutein with anthocyanins showed neither synergy nor antagonism in cellular antioxidant activity (CAA) in Caco-2 cells. Lutein uptake by Caco-2 cells was not significantly altered by the presence of anthocyanins. The maintained intracellular lutein content may partly explain the additive CAA seen in all of the lutein-anthocyanin combinations. It seems that the interaction between anthocyanins and carotenoids on cellular antioxidant activity is partly relevant to the interference of anthocyanins with the cellular uptake of carotenoids. In a previous study, we found that some anthocyanins increase the intracellular content of β-carotene to certain levels where it exerts pro-oxidant activity, which partly explains the observed antagonism of CAA in some of the mixtures [7]. In this study, we found that the cellular uptake of lutein was not affected by the presence of anthocyanins, and the interactive cellular antioxidant effects in all tested lutein-anthocyanin mixtures were additive. The effect of anthocyanins on lutein uptake, however, did not show relevance to the interactive anti-inflammatory effects. The intracellular content of lutein was not significantly changed by the presence of anthocyanins, but some of the lutein-anthocyanin mixtures showed non-additive anti-inflammatory effects on the suppression of interleukin-8 secretion and NO production. This indicates that the combined anti-inflammatory effects between lutein and anthocyanins might not be a consequence of the uptake interaction between the compounds. The synergistic effect of a phytochemical mixture on cellular bioactivities can be the result of the multi-target effects of its phytochemical components on different biomarkers (e.g., oxidative and/or defensive enzymes, inflammatory mediators, gene expression) [6,13]. Molecular mechanisms of anti-inflammatory antagonism between phytochemicals, however, have not been uncovered. There is a limitation of method availability for the prediction of expected gene expressions of inflammatory markers resulting from the combined activity of phytochemicals.

## 3. Materials and Methods

### 3.1. Materials

Lutein (LUT) and six anthocyanidin glucosides: cyanidin-3-*O*-glucoside chloride (CG) (or kuromanin chloride), delphinidin-3-*O*-glucoside cholride (DG) (or myrtillin chloride), pelargonidin-3-*O*-glucoside chloride (PLG) (or callistephin chloride), malvidin-3-*O*-glucoside chloride (MG) (or oenin chloride), peonidin-3-*O*-glucoside chloride (PNG), and petunidin-3-*O*-glucoside chloride (PTG) were ordered from Extrasynthese (Lyon, France). Other chemical reagents were ordered from Sigma Aldrich (Sydney, NSW, Australia). Cell culture medium components were purchased from Gibco^TM^ (Life Technologies, Mulgrave, VIC, Australia).

### 3.2. Phytochemical Stock Preparation

Stocks of anthocyanidin glucosides (1 mg/mL, in methanol) and lutein (1 mg/mL, in tetrahydrofuran) were stored at −80 °C. The concentration of lutein was checked prior to making up working solutions by measuring absorbance at 446 nm (extinction coefficient = 144,500 L/mol·cm^−1^) (UV-1800 series spectrometer, Shimadzu, Tokyo, Japan) [19].

### 3.3. Inhibition of Liposome Peroxidation

The preparation of unilamellar liposomes (0.5 mg/mL) was based on the method of Roberts and Gordon [20] with some modifications described in our previous paper [7]. The final concentration of lutein and/or anthocyanins in liposomes was 0.25% mol/mol lipid. This concentration was selected after preliminary trials (data not shown). At concentrations higher than 0.25%, loss of lutein was seen during the preparation of liposome (i.e., some lutein was visibly retained on the polycarbonate membrane when passing the liposome suspension through the membrane to form unilamellar liposomes). In addition, lutein at 0.25% mol/mol lipid has been reported to be retained more than 80% in liposomes [21]. The liposome suspension then underwent Fe^3+^/ascorbate-induced peroxidation as described previously by Tan et al. [21]. The percentage inhibition of thiobarbituric acid reagent species (%TBARS) was calculated as:

(1) $$\%\mathrm{TBARS}\;=\;\frac{\left(A_{c}{\;-\;A}_{\mathrm{cb}}\right)\;-\;\left(A_{s}{\;-\;A}_{\mathrm{sb}}\right)}{\left(A_{c}{\;-\;A}_{\mathrm{cb}}\right)}\;$$

where: A_cb_, A_sb_: absorbance of non-phytochemical liposomes (control), and liposomes incorporated with phytochemicals, which was measured at 535 nm prior to the induction of peroxidation; A_c_, A_s_: absorbance of non-phytochemical liposomes (control), and liposomes incorporated with phytochemicals, which was measured at 535 nm after lipid peroxidation (60 min, 37 °C).

### 3.4. In Vitro Anti-Inflammatory Assay: Lipoxygenase Inhibition

Lutein working solution was prepared in 0.064 mM ethylenediaminetetraacetic acid (EDTA) containing 0.54% (*v/v*) Tween 80. Anthocyanin working solutions were prepared in 50 mM phosphate buffer (pH 7.4). The final concentration of each phytochemical in the working solution was 0.024 mM. Trials on enzyme reaction kinetics were conducted to determine the optimum enzyme concentration (i.e., 400 U/mL) for maximal enzyme activity (data not presented). The lipid oxidative reaction started by adding an aliquot of the enzyme substrate: linoleic acid (1.25 mM) [17]) into a test tube containing the phytochemicals (final concentration: 0.2–2 μM of lutein and/or 2–12 μM of anthocyanins) and lipoxygenase (400 U/mL). The inhibitory effect of lutein and/or anthocyanins on lipoxygenase activity was assayed according to the protocol of Durak et al. [22] and calculation of the activity was based on the following equation:

(2) $$\mathrm{Inhibition}\;\left(\%\right)\;=\;\frac{\left(A_{c}{\;-\;A}_{\mathrm{cb}}\right){\;-\;(A}_{s}{\;-\;A}_{\mathrm{sb}})}{A_{c}{\;-\;A}_{\mathrm{cb}}}\;$$

where: A_c_: Absorbance of control sample for 100% enzyme activity (no test compounds, added enzyme); A_cb_: Absorbance of control blank (to correct for background absorbance of substrate); A_s_: Absorbance of test sample (added test compounds and enzyme); A_sb_: Absorbance of sample blank for 0% enzyme activity (added test compounds, no enzyme, to correct for background absorbance of the test compounds).

### 3.5. General Cell Culture Conditions

Human Caco-2 cells (passages 45–55) were well maintained in a complete growth medium containing: Dulbecco’s modified eagle medium (DMEM Gibco^TM^, Life Technologies), foetal bovine serum (10%, Bovogen Biologicals, Keilor East, VIC, Australia), GlutaMax^TM^ (1%, Gibco^TM^, Life Technologies), non-essential amino acids (1%, Gibco^TM^, Life Technologies) and anti-microbial agents (penicillin and streptomycin, 1%, Gibco^TM^, Life Technologies). The cells were grown in 25 cm^2^ Corning**^®^** flasks (Corning Inc., New York, NY, USA) in a CO_2_ incubator (Touch 190S, LEEC Limited, Nottingham, UK) at 37 °C and 5% CO_2_, and were routinely subcultured after being confluent at 80%. In every cellular experiment, the cells were seeded at 2.5 × 10^5^ cells/mL and grown for 14 days with a change of medium every day after 100% confluence. The final concentration of lutein or anthocyanins that were loaded onto the cells in each treatment was 2.5–7.5 μM. Tween 40 (0.1% final) was used to deliver lutein into the cells [23].

### 3.6. Cell Viability

A MTT (3-(4,5-dimethylthiazol-2-yl)-2,5-diphenyltetrazolium bromide) (Invitrogen^TM^, Life Technologies) assay described in our previous paper [7] was used to test the cytotoxicity of lutein and anthocyanins (2.5–10 μM).

### 3.7. Cellular Antioxidant Assay

A cell-based assay for testing the antioxidant activity of phytochemicals was adopted from Wolfe and Liu [24] with some modifications as described in our previous paper [7]. Cellular antioxidant activity (CAA) was determined as:

(3) $$\;\mathrm{CAA}\;\mathrm{unit}\;=\;100-\frac{{\mathrm{AUC}}_{s}}{{\mathrm{AUC}}_{c}}\;\times \;100\;$$

where:

- AUC_s_ is the integrated area under the sample fluorescence versus time curve;
- AUC_c_ is the integrated area under the control fluorescence versus time curve;
- Fluorescence excitation was measured at 485 nm and fluorescence emission was measured at 520 nm every 5 min for 12 cycles at 37 °C.

### 3.8. TNF-α-Induced Inflammation

A modified protocol adopted from Peng et al. [25] was used. Caco-2 cells were seeded on 48-well plates (Corning COSTAR**^®^**, Corning Inc.) for 14 days, and subsequently treated with 200 μL lutein and/or anthocyanins (2.5–7.5 μM) at 37 °C and 5% CO_2_. Human TNF-α (50 μL, 500 ng/mL) (Gibco^TM^, Life Technologies) was added to the cells in each well to induce inflammation for 24 h. The cell supernatants were analysed for interleukin-8 (IL-8) and nitric oxide secretion. A human IL-8 ELISA kit (BD OptEIA^TM^, BD Biosciences, San Diego, CA, USA) was used to determine IL-8 concentration, and a Griess reagent kit (Invitrogen^TM^, Life Technologies) was used to measure total nitric oxide (as nitrite) produced by the cells. Vanadium chloride (VCl_3_) (8 mg/mL in 1 M HCl) was used to convert nitrate to nitrite [26].

### 3.9. Cellular Uptake of Lutein

Caco-2 cells were seeded on 6-well plates (Corning COSTAR**^®^**, Corning Inc.) for 14 days and subsequently treated with 2 mL lutein and/or anthocyanins (2.5–7.5 μM) for 4 h at 37 °C, 5% CO_2_. The cells were rinsed with 2 mL cold Dulbecco’s Phosphate-Buffered Saline (DPBS) containing 0.1% Tween 40 followed by a wash with 2 mL pure DPBS. The cells were lysed in 3 mL of cold water for 30 min [27]. The cell lysate was used immediately for lutein extraction.

### 3.10. Extraction of Lutein from Cell Lysate

A modified protocol of carotenoid extraction from cell lysates adopted from Biehler et al. [27] was used. In brief, the cell lysate was mixed with 0.1% butylated hydroxytoluene (BHT)-containing hexane:ethanol:acetone (2:1:1, *v*/*v*/*v*, 4 mL) and an aliquot of the internal standard: *trans*-β-Apo-8′-carotenal (Sigma Aldrich, Sydney, Australia). The tubes were sonicated (2 min) and centrifuged (4000× *g*, 5 min). The hexane phase was collected and a secondary extraction of the cell lysate was carried out with hexane (2 mL, containing 0.1% BHT). The hexane phase was pooled, dried under nitrogen and stored at −80 °C until LC-MS analysis.

### 3.11. Lutein Analysis by LC-MS

Identification and quantification of lutein in the cultured cells was carried out following a method previously developed by our research group [7] on a HPLC system (Accela, Thermo Fisher Scientific Inc., Waltham, MA, USA) connected to a LTQ Orbitrap XL^TM^ mass spectrometer (Thermo Fisher Scientific Inc.) equipped with an atmospheric pressure chemical ionization source (APCI). Lutein extracts and standards (20 μL) were injected into a 2.1 mm × 250 mm C30 column (Acclaim^TM^, 3 μm particle size, Thermo Fisher Scientific Inc.). Mobile phase components and the gradient of mobile phase were given in our previous report [7]. MS instrumental parameters were set as shown in [7]. The identification of lutein was based on its relative retention time and its accurate mass (*m*/*z* 568.43). Extracted ion current chromatograms of *m*/*z* 568.0–568.5 were plotted for the identification and quantification of lutein. Instrument control and data processing were performed using XCalibur^TM^ software (version 2.2, Thermo Fisher Scientific Inc., San Jose, CA, USA).

### 3.12. Mode of Interaction Determination

A comparison between the experimental effect of every mixture with its expected additive effect was made to determine the mode of interaction. An equation given by Fuhrman et al. [12] was used to calculate the expected activity. The mode of phytochemical interaction is defined as:

- Synergy: the experimental inhibitory activity is greater than the expected activity;
- Antagonism: the experimental inhibitory activity is lesser than the expected activity;
- Addition: the experimental inhibitory activity is equal to the expected activity.

### 3.13. Data Analysis

All chemical- and cell-based experiments were conducted at least in triplicate. One-way analysis of variance (ANOVA) and Tukey’s test were performed to compare means for significant difference at *p* < 0.05 using Minitab (version 9.0, Minitab Inc., State College, PA, USA).

## 4. Conclusions

The combinations of lutein and anthocyanins did not show synergistic antioxidant effects in the tested chemical and cellular models. Lutein and anthocyanins (1:1, 2 μM) showed an antagonistic interaction on lipid peroxidation in a phosphatidylcholine liposome membrane. All of the combinations at the tested ratios (1:1, 1:3 and 3:1, total concentration of 10 μM), however, showed additive effects on cellular antioxidant activity in a Caco-2 cell model. The cellular uptake of lutein (2.5–5 μM) was not affected by the presence of anthocyanins (5–7.5 μM), which could partly explain the observed additive cellular antioxidant activity. Only the mixture of LUT with MG showed anti-inflammatory synergy in the suppression of interleukin-8 at all tested ratios. Some lutein-anthocyanin combinations showed antagonism in the suppression of pro-inflammatory mediators (IL-8, NO) despite the fact that at the concentrations tested, lutein uptake was not affected by the presence of anthocyanins. Future studies should be designed to unravel the molecular mechanisms of anti-inflammatory antagonism of mixed phytochemicals. An understanding of phytochemical combinations and the appropriate concentrations can lead to designing foods or supplements with better targeted functions and absorption.

## Figures and Tables

**Table 1:** Cellular antioxidant activity (CAA) of lutein-anthocyanin mixtures.

Mixture	Lutein: Anthocyanin Ratio					
	1:3		1:1		3:1	
	Experimental Effect ^1^	Expected Additive Effect ^2^	Experimental Effect	Expected Additive Effect	Experimental Effect	Expected Additive Effect
LUT-CG	38.1 ± 12.5	52.1	36.9 ± 12.8	52.1	46.3 ± 7.5	45.2
LUT-DG	48.2 ± 12.3	49.4	45.5 ± 5.9	36.4	44.1 ± 5.3	44.0
LUT-MG	50.8 ± 7.1	48.1	44.0 ± 3.1	42.5	50.3 ± 6.8	50.4
LUT-PNG	42.9 ± 6.9	43.9	33.6 ± 8.8	38.1	48.7 ± 2.4	46.8
LUT-PLG	38.6 ± 4.8	44.3	32.5 ± 8.6	38.9	49.2 ± 4.0	45.8
LUT-PTG	38.2 ± 6.5	42.6	34.6 ± 11.5	39.2	47.6 ± 5.9	45.7

LUT: lutein, CG: kuromanin chloride, DG: myrtillin chloride, MG: oenin chloride, PNG: peonidin-3-glucoside chloride, PLG: callistephin chloride, PTG: petunidin-3-glucoside chloride. ^1^ Each value of the experimental effect was mean of CAA unit ± SD of four individual replicates. ^2^ The expected additive effect was calculated as CAA_A_ + CAA_L_ − CAA_A_ × CAA_L_/100 (CAA_A_ and CAA_L_ is the CAA unit of an anthocyanin alone and lutein alone respectively).
